# Effects of Astaxanthin from *Litopenaeus Vannamei* on Carrageenan-Induced Edema and Pain Behavior in Mice

**DOI:** 10.3390/molecules21030382

**Published:** 2016-03-19

**Authors:** Zulkiflee Kuedo, Anantita Sangsuriyawong, Wanwimol Klaypradit, Varomyalin Tipmanee, Pennapa Chonpathompikunlert

**Affiliations:** 1 Department of Physiology, Faculty of Science, Prince of Songkla University, Hat Yai, Songkhla 90112, Thailand; lee.kuedo@gmail.com; 2 Center for Advanced Studies for Agriculture and Food, Kasetsart University, Bangkok 10900, Thailand; anantita_sw@hotmail.com (A.S.); ffiswak@ku.ac.th (W.K.); 3 Department of Biomedical Sciences, Faculty of Medicine, Prince of Songkla University, Hat Yai, Songkhla 90110, Thailand; tvaromya@medicine.psu.ac.th

**Keywords:** astaxanthin, *Litopenaeus vannamei*, inflammatory pain, carrageenan, myeloperoxidase, mice

## Abstract

Carrageenan produces both inflammation and pain when injected in mouse paws via enhancement of reactive oxygen species formation. We have investigated an effect of astaxanthin extracted from *Litopenaeus vannamei* in carrageenan-induced mice paw edema and pain. The current study demonstrates interesting effects from astaxanthin treatment in mice: an inhibition of paw edema induced in hind paw, an increase in mechanical paw withdrawal threshold and thermal paw withdrawal latency, and a reduction in the amount of myeloperoxidase enzyme and lipid peroxidation products in the paw. Furthermore the effect was comparable to indomethacin, a standard treatment for inflammation symptoms. Due to adverse effects of indomethacin on cardiovascular and gastrointestinal systems, our study suggests promising prospect of astaxanthin extract as an anti-inflammatory alternative against carrageenan-induced paw edema and pain behavior.

## 1. Introduction

Inflammation is a cellular response to a traumatic injury, irritation from toxic chemicals, or infection caused by microbial pathogens. This complex process involves numerous pathways of cellular and plasma origin with interrelated biological events, often leading to painful conditions, such as in rheumatoid arthritis, asthma, allergy, inflammatory bowel syndrome, atherosclerosis, and neurodegenerative disease [1,2]. The phenomena of inflammations are largely regulated by various mediators like reactive oxygen and nitrogen intermediates, prostacyclins, prostaglandin, leukotrienes, cytokines, and histamine, which could be expressed in macrophages, hepatocytes, and endothelial or smooth muscle cells [3]. One of distinctive mediators is nitric oxide radical, generated from the terminal guanido-nitrogen atom of l-arginine by NADPH-dependent enzymes known as nitric oxide synthase (NOS), of which the Ca^2+^ independent inducible form (iNOS) has been expressed in inflamed tissues in response to lipopolysaccharide (LPS), interferon-gamma (INF-γ), tumor necrosis factor (TNF), interleukin-1beta (IL-1β), and found to be responsible for the pathophysiology of inflammation [4]. The highly reactive nitric oxide radical could be initiated form another stronger oxidant, *viz*. peroxynitrite (ONOO^−^) by combining with superoxide anion radicals, harmful to functional normal tissues [5,6]. This aforementioned statement reflects the role of reactive oxygen and nitrogen species in the pathophysiology of inflammation. In addition scavenging of superoxide radicals and suppression of nitric oxide and iNOS protein could represent a novel therapeutic approach against inflammatory diseases.

In general, steroids and cyclooxygenase inhibitors such as prednisolone, aspirin, and indomethacin have long been used as the main therapeutic anti-inflammatory agents, but they are frequently associated with significant detrimental effects in patients especially gastrointestinal toxicity [7,8]. Non-steroidal anti-inflammatory drugs (NSAIDs) clearly promote reactive oxygen species (ROS) production. It was proposed that NSAID-mediated gastrointestinal lesions involve the uncoupling of oxidative phosphorylation and inhibition of the electron transport chain causing incomplete reduction of oxygen. Indomethacin, a potent NSAID, was found to bind to a site near complex I and ubiquinone to generate ROS [9,10], and ROS can damage cellular lipids, proteins, and DNA, leading to oxidative stress [11]. Potentially developing natural alternative anti-inflammatory supplements have, thus, increasingly become important [12]. This approach seemingly overcomes the incidences of drug related toxicity and iatrogenic reactions caused by 90% of the non-steroidal anti-inflammatory drugs (NSAIDs) commonly used for treatment of inflammatory conditions [13]. Hence, it would be interesting to explore some of the exotic dietary ingredients customary in ethnic cultures around the world so that the natural product with prospective anti-inflammatory properties could be well-documented and validated scientifically.

Many phytochemicals, particularly carotenoids and flavonoids, are well-known of capability in cellular redox imbalance modulation, as well as the endothelial and metabolic processes regarding the pathogenesis of inflammatory. Astaxanthin is one of the most common carotenoids and found in the red pigment in crustacean shells (crabs, shrimps, for example), salmon, and Asteroidean [14]. Moreover, astaxanthin, as a nutraceutical, has been reported to possess the potential preventive capacity associated with health benefits [15] partly due to its free radical antioxidant activity up to 100-fold stronger than vitamin E [16]. Additionally, previous studies demonstrated that astaxanthin intake as a nutritional supplement can prevent oxidative damages resulting in a decrease in some chronic diseases [17,18,19], exhibit anti-tumor activity [20], inhibit proliferation of breast and colon cancer [21,22], and reduce significantly chronic inflammatory diseases [23,24,25]. Since the common source, like *Haematococcus pluvialis*, is not able to be cultivated in Thailand, an interesting alternative source of astaxanthin is *Litopenaeus vannamei*, which plays an enormous role in national exportation. Large amount of crustacean shells are however wastefully left out. Additionally, any scientific supports for astaxanthin from have not yet been established. Herein the present study reported the anti-inflammatory activities of astaxanthin extracted from the white shrimp shell *(Litopenaeus vannamei)* mostly found in Thailand.

## 2. Results

### 2.1. Effects of Astaxanthin on Carrageenan-Induced Paw Edema

**Figure 1:**
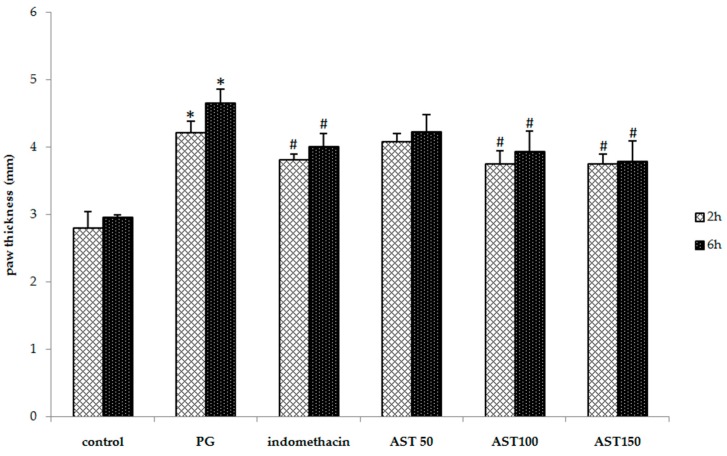
Effect of astaxanthin (AST) on carrageenan-induced paw edema (mm). Data are presented as mean ± SD. * *vs.* naïve control, *p* < 0.05 with respect to identical time; # *vs.* vehicle control (propylene glycol or PG), *p* < 0.05 with respect to identical time; *n* = 8 mice/group.

As shown in Figure 1, indomethacin (5 mg/kg), a standard anti-inflammatory drug, and astaxanthin at a dose of 100–150 mg/kg significantly suppress the carrageenan-induced increases in paw thickness (3.81 ± 0.08, 3.76 ± 0.19, 3.75 ± 0.29 mm at 2 h and 4.01 ± 0.19, 3.93 ± 0.30, 3.79 ± 0.13 mm at 6 h, respectively) compared to propylene glycol-treated animals (vehicle controls) (4.22 ± 0.15 mm) at 2 h and (4.65 ± 0.21 mm) at 6 h after the injection. Although a significant increase in paw thickness was observed in all groups, the mice that received indomethacin or astaxanthin displayed significantly less edema. Furthermore, astaxanthin (100–150 mg/kg) displayed a similar efficacy to indomethacin in the suppression of inflammation-induced increase in paw thickness. However, a low dose of astaxanthin (50 mg/kg) showed no significant effect (Figure 1). The ineffectiveness of this low dose may be due to its insufficient therapeutic dose to express the protective effect for an inflammation.

### 2.2. Effects of Astaxanthin on Carrageenan-Induced Hyperalgesia

**Figure 2:**
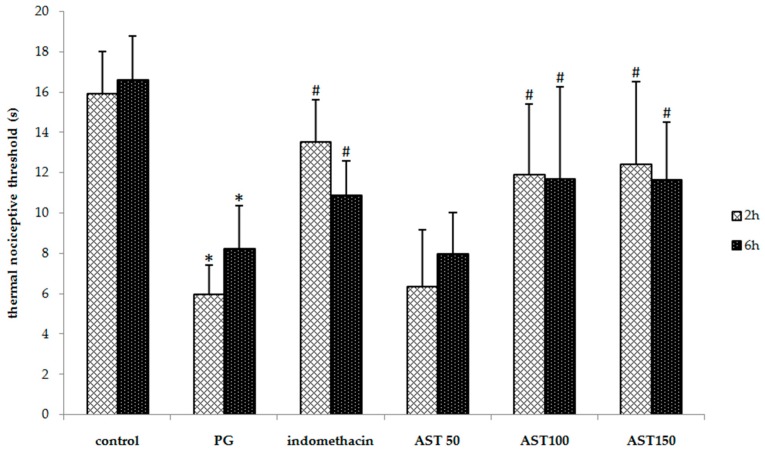
Effect of astaxanthin (AST) on carrageenan-induced thermal hyperalgesia. Data are presented as mean ± SD. * *vs.* naïve control, *p* < 0.05 with respect to identical time; # *vs.* vehicle control (propylene glycol or PG), *p* < 0.05 with respect to identical time; *n* = 8 mice/group.

**Figure 3:**
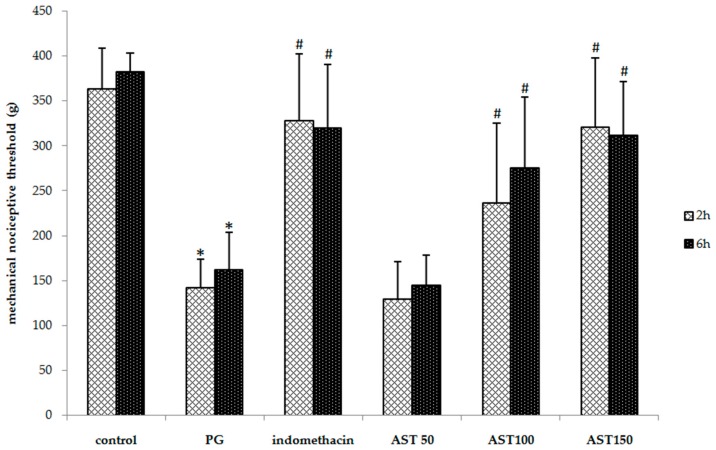
Effect of astaxanthin (AST) on carrageenan-induced mechanical hyperalgesia. Data are presented as mean ± SD. * *vs.* naïve control, *p* < 0.05 with respect to identical time; # *vs.* vehicle control (propylene glycol or PG), *p* < 0.05 with respect to identical time; *n* = 8 mice/group.

Each mouse received propylene glycol, astaxanthin (100–150 mg/kg) or indomethacin (5 mg/kg) via oral gavage, directly followed by a hind paw injection of 2.5% carrageenan (50 µL). A one-way ANOVA revealed that the carrageenan-induced thermal and mechanical threshold was significantly suppressed by indomethacin and astaxanthin (*P* < 0.05). *Post hoc* statistical testing showed that the paw withdrawal latency and mechanical paw withdrawal threshold of animals treated with astaxanthin or indomethacin were significantly greater than which of vehicle-treated mice at 2 and 6 h after the carrageenan injection (Figure 2 and Figure 3). In addition, no significant difference between the astaxanthin (100–150 mg/kg) and indomethacin-treated animals was observed. These results indicate that astaxanthin was as effective as indomethacin in reducing inflammation-induced thermal and mechanical hyperalgesia. Note that a dose of 50 mg/kg was not effective to decrease hyperalgesia.

### 2.3. Effects of Astaxanthin on Carrageenan-Induced MPO Accumulation

**Figure 4:**
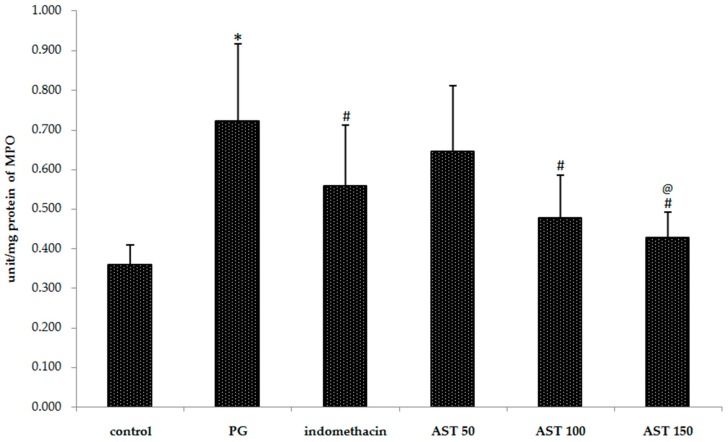
Effect of astaxanthin (AST) on carrageenan-induced MPO accumulation. Data are presented as mean ± SD. * *vs.* naïve control, *p* < 0.05; # *vs.* vehicle control (propylene glycol or PG), *p* < 0.05; @ *vs.* indomethacin, *p* < 0.05; *n* = 8 mice/group.

Indomethacin and astaxanthin (100–150 mg/kg) significantly reduced the neutrophil content, evaluated from myeloperoxidase activity, in the carrageenan*-*induced inflammation. The myeloperoxidase concentration (*i.e.*, the neutrophil recruitment) in the hind paw of indomethacin, astaxanthin 100 mg/kg and astaxanthin 150 mg/kg treated mice were 0.558 ± 0.154, 0.478 ± 0.106, and 0.429 ± 0.063 units/mg protein respectively, compared to 0.722 ± 0.195 units/mg protein in the vehicle controls (*p* < 0.05) (Figure 4), whereas 50 mg/kg had given an unsatisfying result (0.645 ± 0.166 units/mg protein).

### 2.4. Effects of Astaxanthin on Carrageenan-Induced ROS Release

**Figure 5:**
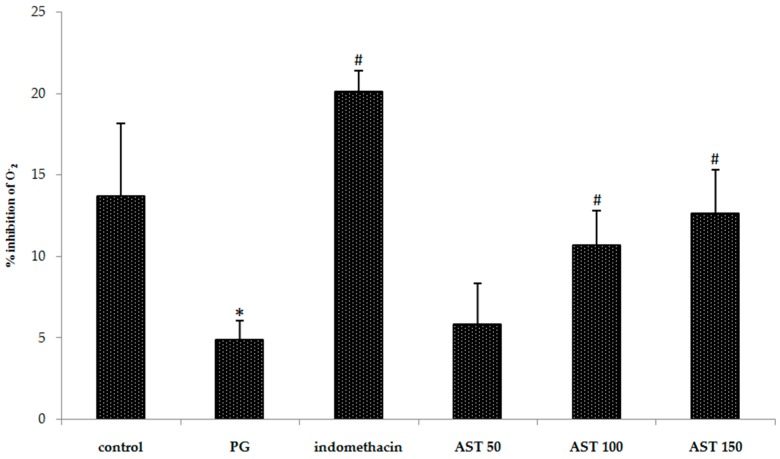
Effect of astaxanthin (AST) on carrageenan-induced ROS release. Data are presented as mean ± SD. * *vs.* naïve control, *p* < 0.05; # *vs.* vehicle control (propylene glycol or PG); *n* = 8 mice/group.

**Figure 6:**
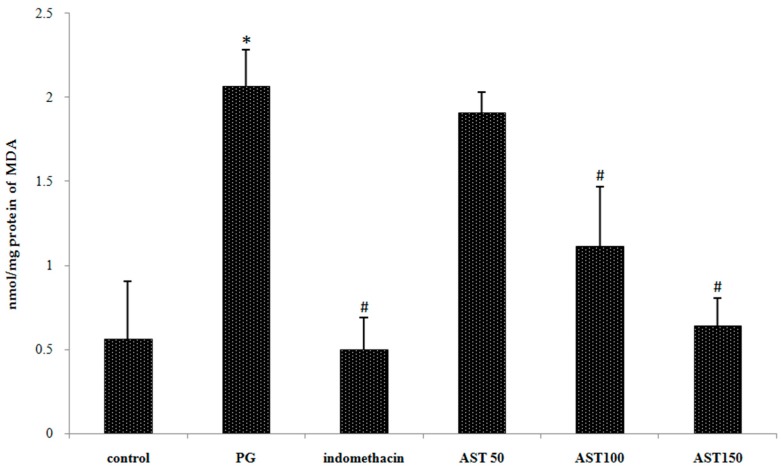
Effect of astaxanthin (AST) on carrageenan-induced lipid peroxidation product or MDA. Data are presented as mean ± SD. * *vs.* naïve control, *p* < 0.05; # *vs.* vehicle control (propylene glycol or PG); *n* = 5 mice/group.

In order to evaluate free radical level, inhibition percentage of superoxide anion and malondialdehyde (MDA) level were measured. We found that carrageenan-treated groups show significantly increased superoxide anion level and MDA level in paw tissue. Indomethacin and astaxanthin (100–150 mg/kg) treated group showed significant effect to attenuate superoxide anion and MDA increase after carrageenan injection in paw tissue (Figure 5 and Figure 6). A low dose (50 mg/kg) displayed an insignificantly different effect compared to PG group.

## 3. Discussion

The most common test to screen new anti-inflammatory agents in order to elucidate the ability of a compound to reduce local edema induced is to inject an irritant agent into the rodent paw [26]. Carrageenan-induced edema has been widely used as an experimental animal model for acute inflammation and is believed to be biphasic. An initial phase lasting up to 2 h is primarily mediated via rapid production of inflammatory mediators such as histamine, serotonin, bradykinins, and increased synthesis of prostaglandins in the damaged tissue surroundings [27,28,29,30]. In contrast, the late phase lasting from 3–6 h is sustained by prostaglandin release from macrophages and mediated by bradykinin, leukotrienes, polymorphonuclear cells, mobilized phagocytic cells, monocytes, macrophages, ROS, nitric oxide, proteolytic enzymes, and platelet activating factor [27,29,30,31,32,33]. The present study showed that astaxanthin extract, via oral administration, simultaneously inhibited both phases in the carrageenan-induced paw edema test (Figure 1). These reports supported that the time period for induced inflammatory response of carrageenan displayed different severity which is consistent with previous studies [34,35].

Increased pain sensitivity is a common feature of the inflammatory response and occurs after tissue injury. The peripheral sensitization is triggered by NF-κB-related pro-inflammatory mediators, including the cytokines TNF-α and IL-1β [36,37,38], as well as ROS, such as the superoxide anion radical [39]. In addition to their role in the inflammatory response, these mediators can act directly on their receptors or targets expressed by peripheral terminals of nociceptors to reduce pain thresholds, leading to inflammatory pain and hyperalgesia [40,41]. Moreover, an induction of neutrophils recruitment leads to the further enhancement of hyperalgesic mediators, such as prostaglandin E_2_ (PGE_2_) and the superoxide anion [36,39]. Previous studies also reported that carrageenan is a known toxic substance to induce hyperalgesia in rodents [42,43]. These reports were corresponding to a mode of action shown in our data that carrageenan can decrease paw withdrawal latency and mechanical paw withdrawal threshold when compare to the vehicle control group. Interestingly, astaxanthin extract at a dose of 100–150 mg/kg via the oral route can attenuate and decreased both thermal and mechanical nociceptive threshold tests (Figure 2 and Figure 3). This emphasizes that our astaxanthin extract therefore diminished the carrageenan-induced hyperalgesia. Moreover, previous study found that astaxanthin can inhibit ROS-induced production of NF-κB transcription factor, which in turn effectively inhibits the production of inflammatory cytokines [44]. So, astaxanthin extract may decrease pain via inhibiting the pro-inflammatory cytokine mediator.

Since neutrophil recruitment is an important mediator of inflammation, and the enzyme MPO is a reliable marker for the detection of neutrophil accumulation in inflamed skin *in vivo* [45], we have measured the MPO activity in the mouse paw injected with carrageenan. The results show that astaxanthin extract could decrease its activity (Figure 4). Hence, it will be reasonable to speculate that an inhibition of MPO occurred through the neutrophil modulation, which could partially account, at least, for the anti-inflammatory properties of astaxanthin.

Oxidants are well-known in playing a significant role in the pathogenesis of a number of disorders such as inflammation. Oxidative stress is defined as an imbalance between cellular production of ROS and antioxidant defense mechanisms. ROS (e.g., superoxide radical, peroxynitryl, hydroxyl radical, and hydrogen peroxide) are key signaling molecules in the progression of inflammatory disorders. An enhanced ROS generation by polymorphonuclear neutrophils (PMNs) at the site of inflammation causes endothelial dysfunction and tissue injury [46,47]. However, superoxide anion level cannot be directly measured. To evaluate the level, the measurement of superoxide anion inhibition was instead applied. In our experiment, carrageenan-induced model can successfully produce ROS (Figure 5 PG group), similar to previously reported experiments [48]. Herein MDA was chosen as a biomarker to confirm ROS occurrence because ROS can damage cellular tissue, and plasma membrane, leading to a product due to lipid peroxidation called MDA [49,50]. Thus, inflammatory response would cause the accumulation of MDA. Interestingly our results indicated that the MDA production was reduced by treatment of astaxanthin extract (Figure 6). The results likely provide information regarding anti-inflammatory property of astaxanthin extract which is in accordance with the published study concerning astaxanthin as a free radical scavenger via singlet oxygen quenching [51]. Apart from MDA production, we also evaluate an anti-inflammatory property of astaxanthin by a comparison to a case study of superoxide dismutase (SOD) enzyme. Typically, SOD is a scavenging enzyme responsible for free radical regulation. The use of poly(ethylene glycol)-linked human recombinant SOD suggested that the generation of superoxide anions, together with nitric oxide, and peroxynitrite, involves in the acute carrageenan-induced paw edema [33,52]. Early clues of evidence pointed for the implication of superoxide anion in acute carrageenan-induced inflammation [53], as well as in ischemic paw edema [54]. A study of SOD mimetic M40403, implicated a key role for superoxide anion in inflammatory pain [39]. In this study, it is noteworthy that astaxanthin extract from *Litopenaeus vannamei* (100–150 mg/kg), where all experiments were performed using carrageenan-induced inflammation, display inhibitory effects against superoxide anion radical (Figure 5) similar to the earlier result [55]. Our study was adequate to imply active SOD-like potency.

As we have known that indomethacin can act as an inhibitor of prostaglandin production via inhibition of cyclooxygenase 1 and 2 enzymes in the late phase of carrageenan-induced inflammation [56,57], our results show that astaxanthin extract exhibit an efficacy (100–150 mg/kg) comparable to indomethacin (5 mg/kg) in all experiments including edema, thermal and mechanical hyperalgesia, inhibition percentage of superoxide anion, MDA level, and MPO activity. However, the therapeutic effect of indomethacin is stronger, if one considered at the same dose. Although indomethacin demonstrated efficacy in pain relief and inflammation [58], indomethacin is found to be associated with a lot of numbers of adverse effects, for example, alterations in renal function, effects on blood pressure, hepatic injury and platelet inhibition which may result in increased bleeding. In addition, the most important adverse effects of indomethacin are the gastrointestinal and cardiovascular adverse effects, respectively [59]. This leaves an opportunity for astaxanthin extract to be an interesting choice for pain control from inhibits inflammation process.

Previous studies reported that an astaxanthin extract from *Aristeus alcocki* (Arabian red shrimp) shell waste is a more powerful antioxidant than one in the *Haematococcus pluvialis* [60]. A higher proportion of astaxanthin diester and a higher content of poly unsaturated fatty acids (20% PUFAs in monoester and 30% PUFAs in diester) in the carotenoid extract obtained from *Aristeus alcocki* shell is suggested to be responsible for the distinctive antioxidant property. Significant evidence supports the versatile properties of astaxanthin as a neutraceutical, such as protective effect from oxidative stress in healthy smokers [17], tumor prevention in breast cancer [21], oral cancer [61] and colon cancer [22], and cardiovascular disease [62], as well as neurodegenerative disease and metabolic syndromes [63]. Interestingly, our astaxanthin extract from *Litopenaeus vannamei* (Pacific white shrimp) shell waste contains PUFA content of 61.74% [64], leading to an implication that the powerful antioxidant property of carotenoid extract of *Litopenaeus vannamei* may be attributed to the antioxidant synergism of astaxanthin and PUFAs present in the extract. Moreover, free astaxanthin that accounted from *Litopenaeus vannamei* also showed a large percentage (32.95%), different from the pattern of *Haematococcus pluvialis* (5%) [65]. Although the effect of astaxanthin from *Aristeus alcocki* (Arabian red shrimp) shell waste can inhibit carrageenan-induced inflammation in mice [60], the source of astaxanthin extract and route of administration are different from our study. To sum up, our study offers a new source of astaxanthin with a better potency to protect inflammation.

## 4. Materials and Methods

### 4.1. Animals

Male ICR mice, eight weeks old, weighting 30–35 g, were acclimated to housing for at least one week prior to investigation. Mice were on a 12/12-h day/night cycle, a relative humidity of 50%, with food and water provided *ad libitum*. All administrations in this study were performed once daily between 8:00–9:00 a.m. Six groups of ICR mice (*n* = 8) were randomly assigned into naïve control, vehicle control (propylene glycol treated groups), positive control (indomethacin treated groups), and treatment groups (50, 100, 150 mg/kg of astaxanthin). Experimental procedures have been performed in accordance with the principles of animal care outlined by Faculty of Science, Prince of Songkla University (Ethic No. MOE 0521.11/019). The studies were performed according to the guidelines of the Committee for Research and Ethical Issues of the International Association for the Study of Pain [66].

### 4.2. Model of Inflammation

In this model, each mouse was injected with 50 µL of the inflammatory agent under brief isoflurane anesthesia (3%). To assess the effects of astaxanthin from the white shrimp shell on paw edema, 2.5% carrageenan suspension (*w*/*v* with 0.9% saline) was injected into both hind paws.

### 4.3. Extraction of Astaxanthin from White Shrimp Shell

Fresh white shrimp (*Litopenaeus vannamei*) shells were obtained from a frozen shrimp processing plant in Samut-Sakorn province, Thailand. The samples were packed and stored frozen at −20 °C. Prior to use, samples were thawed by submerging samples under cool running tap water. Astaxanthin extraction was prepared according to the procedure described by Sachindra, Bhaskar, and Mahendrakar [67] with slight modification in that food-grade ethanol was used as a solvent. Shrimp shell was blended with ethanol in the ratio of 1:2 (shrimp shell:ethanol) using a Waring laboratory blender (Waring Laboratory Science, Winsted, MN, USA). Shrimp shell residues were vacuum filtered. The collected extract was then evaporated under vacuum at 40 °C, 175 MPa using a Büchi R-124 rotary evaporator (Büchi Labortechnik AG, Flawil, Switzerland) to obtain astaxanthin. Herein, extraction yield was 20.39 ± 1.02 mg of astaxanthin/g shrimp shell.

### 4.4. Measurement of Paw Edema

Paw thickness was used as a measurement of inflammation-induced edema [26,27,28,29,30,31]. Briefly, the dorsoventral thickness of each hind paw was measured using a caliper placed at the border of the phalanges and the metatarsals. The measurement was taken when each edge of the caliper was just touching the dorsal and ventral surface of the hind paw (*i.e.*, the caliper was not squeezed onto the hind paw). Data are expressed as the mean paw thickness ± SD.

### 4.5. Behavioral Assessment of Thermal Nociceptive Threshold Test

Animals were acclimated to the laboratory environment, investigator handling and behavioral equipment during at least two training sessions. A single investigator injected all animals and collected all behavioral data. To blind the investigator during the behavioral measurements, multiple substances doses were used in each experimental session and the animals were randomly placed onto the testing apparatus. The room temperature of the behavioral testing facility was maintained at 3 ± 2 °C.

The unilateral hot plate test (the plantar side of one hind paw is placed on a hot plate surface and, thus, the withdrawal latency of each paw can be measured separately) was employed to measure central analgesic activity by the method of Menѐndez *et al.*, with modifications from Hargreaves *et al.* [68,69]. Thermal paw withdrawal latencies (PWL) to radiant heat stimuli were recorded from the left and right hind paw of each mouse. Mice were placed into plexiglass chambers on a glass surface that was heated to 45 ± 1 °C. Following a 10 min acclimation period, a radiant heat stimulus was alternately applied to each hind paw, and the time to paw withdrawal measured. Each hind paw was tested two times. A 20 s exposure limit was imposed to prevent tissue damage, and a 5 min interval was maintained between trials to avoid sensitization. The PWL was calculated as the mean of two trials from both paws, and data across animals are presented as the mean ± S.D.

### 4.6. Behavioral Assessment of Mechanical Nociceptive Threshold Test

The mechanical nociceptive withdrawal threshold was assessed by using the Randall-Selitto electronic algesimeter (Ugo basile, Analgesy-Meter Cat. No. 37215, Italy). Before the test, each animal received 5 min of handling to get used to manipulation. The tested paw will be placed between a flat surface and wedge-shaped blunt piston, an increasing mechanical force will be applied via a piston onto the dorsal surface of the paw. When the mouse experiences pressure on the paw, it makes reflex movement of withdrawal. Immediately, the application of pressure will be stopped and the threshold (weight in grams) is recorded. The maximum force applied was limited to 200 g to avoid any tissue damage [70].

### 4.7. Myeloperoxidase (MPO) Assay

To determine the recruitment of neutrophils in response to the carrageenan induced inflammation in the mouse hind paw, the paws were weighed and homogenized in cold 50 mM phosphate buffer pH 6 involved 0.5% hexadecyltrimethyl-ammonium bromide (Sigma Chemical Co., St. Louis, MO, USA). Supernatants were collected by centrifugation for 10 min at 10,000 rpm at 4 °C and kept at −80 °C and freeze-thawed, whereafter the myeloperoxidase activity of the supernatant was assessed. The enzyme reaction was carried out in a 96-well plate by adding 190 μL of 50 mM phosphate buffer pH 6, 5 μL of 0.5% o-dianisidine hydrochloride, 5 μL of 20 mM H_2_O_2_ and 10 μL of supernatant sample. After keeping reaction for 30 min at room temperature, the absorbance at 460 nm was measured using a microplate reader (ASYS UVM 340, Biochrom Ltd., Cambridge, UK). MPO activity was determined by using the curve from the standard MPO (Sigma Chemical Co.). Values are expressed as MPO units/mg protein.

### 4.8. Superoxide Anion Assay

Superoxide anions are particularly important as the product of the one-electron reduction of dioxygen superoxide anions, which are widely produced in large quantities by the enzyme NADPH oxidase for use in oxygen-dependent killing mechanisms of invading pathogens but superoxide is biologically toxic. The superoxide anion level was determined by spectrophotometric measurement using a microplate reader (ASYS UVM 340, Biochrom Ltd.). The method based on xanthine/xanthine oxidase (XO) system which converted nitro blue tetrazolium (NBT)-yellow color to formazan-blue color. The reagent mix was prepared of 0.3 mM EDTA, 0.6 mM NBT, 0.1 mM xanthine and 0.02 U/mL XO, mixed with sample and measured at 560 nm compared with standard curve of 4-hydroxy-2,2,6,6-tetramethylpiperidin-1-oxyl (TEMPOL). The data were expressed as % inhibition which was calculated following the equation [71].

% inhibition = (A − B)/A × 100(1)

A = OD of reagent only; B = OD of sample.

### 4.9. Measurement of Lipid Peroxidation

Lipid peroxidation was evaluated by measuring the amount of MDA, following the method of Uchiyama and Mihara [72]. Samples of intestinal tissue from treated mice were collected, homogenized in cold 0.1 mM phosphate buffer (pH 7.4), and centrifuged at 10,000 rpm at 4 °C for 15 min. An aliquot of the supernatant was added to the reaction mixture containing 8% (*w*/*v*) sodium dodecyl sulfate, 20% (*v*/*v*) acetic acid, 0.8% (*w*/*v*) thiobarbituric acid, and distilled water. After incubation at 95 °C for 1 h, the amount of MDA formed in the reaction mixture was measured using a microplate reader (ASYS UVM 340, Biochrom Ltd.) at an absorbance of 532 nm. 1,1,3,3-tetramethoxypropane was used as the standard. The protein concentration of the supernatant sample was measured using the BCA kit as well as the MPO assay.

### 4.10. Experimental Design

Astaxanthin were delivered as a solution with propylene glycol. Indomethacin (Wako Pure Chemical Industries, Ltd., Saitama, Japan), a potent non-steroidal anti-inflammatory agent, was suspended in a propylene glycol. Indomethacin (5 mg/kg) has been reported to reduce pain and inflammation associated with acute inflammation [73,74] and serves as a positive control in this study. Propylene glycol was used as a vehicle control.

Mice were randomly separated to each treatment group (*n* = 8 mice per group). Mice received astaxanthin at various doses ranging from 50–100 mg/kg once daily for three weeks and received indomethacin at a daily dose of 5 mg/kg for one week before carrageenan-induced paw edema. At the last dose of oral administration with astaxanthin (50–100 mg/kg), indomethacin (5 mg/kg), or propylene glycol, each animal received the intraplantar injection of 2.5% carrageenan (50 µL) from Wako Pure Chemical Industries, Ltd. Saitama, Japan. The paw edema testing was performed 2 and 6 h after the carrageenan injection.

### 4.11. Statistical Analyses

All data were presented as the means ± SD. Differences between the mean values for individual groups were assessed by a one-way analysis of variance (ANOVA), followed by LSD’s *post hoc* test. *p* < 0.05 was considered to indicate a statistically significant difference. The SPSS version 13 (IBM Corp., Armonk, NY, USA) was used for the analysis.

## 5. Conclusions

Astaxanthin extract from *Litopenaeus vannamei* have well suppressed nociceptive behaviors associated with acute inflammation and provided a beneficial role in the treatment of inflammatory pain. Also, the results support that astaxanthin can be applied for anti-inflammatory conditions. However, further research is necessary to improve the efficacy and stability of astaxanthin extract, as well as more clinical investigations are required to substantiate this report.
